# Mycodegradation of low-density polyethylene by *Cladosporium sphaerospermum*, isolated from platisphere

**DOI:** 10.1038/s41598-024-59032-4

**Published:** 2024-04-09

**Authors:** M. Sathiyabama, R. V. Boomija, T. Sathiyamoorthy, N. Mathivanan, R. Balaji

**Affiliations:** 1 Department of Botany, School of Life Sciences, Bharathidasan University, Tiruchirappalli, Tamil Nadu 620 024 India; 2 CAS in Botany, University of Madras, Chennai, Tamil Nadu 600025 India

**Keywords:** Mycodegradation, LDPE, Weight loss, Surface erosion, *Cladosporium sphaerospermum*, Microbiology, Environmental sciences

## Abstract

Plastic accumulation is a severe threat to the environment due to its resistivity to thermal, mechanical and biological processes. In recent years, microbial degradation of plastic waste disposal is of interest because of its eco-friendly nature. In this study, a total of 33 fungi were isolated from the plastisphere and out of which 28 fungal species showed halo zone of clearance in agarized LDPE media. The fungus showing highest zone of clearance was further used to evaluate its degradation potential. Based on morphological and molecular technique, the fungus was identified as *Cladosporium sphaerospermum.* The biodegradation of LDPE by *C. sphaerospermum* was evaluated by various methods. The exposure of LDPE with *C. sphaerospermum* resulted in weight loss (15.23%) in seven days, higher reduction rate (0.0224/day) and lower half-life (30.93 days). FTIR analysis showed changes in functional group and increased carbonyl index in LDPE treated with *C. sphaerospermum*. SEMimages evidenced the formation of pits, surface aberrations and grooves on the LDPE film treated with the fungus whereas the untreated control LDPE film showed no change. AFM analysis confirmed the surface changes and roughness in fungus treated LDPE film. This might be due to the extracellular lignolytic enzymes secreted by *C. sphaerospermum* grown on LDPE. The degradation of polyethylene by Short chain alkanes such as dodecane, hexasiloxane and silane were identified in the extract of fungus incubated with LDPE film through GC–MS analysis which might be due to the degradation of LDPE film by *C. sphaerospermum.* This was the first report on the LDPE degradation by *C. sphaerospermum* in very short duration which enables green scavenging of plastic wastes.

## Introduction

Plastics have become an inevitable material in our lives, and it plays a significant role in the global economy^1^. Plastic production increased over time, and approximately three hundred and twenty million tons of plastics are produced every year^2,3^. Polyethylene is the prominent plastic widely used in various industries. High-density polyethylene (HDPE) and Low-density polyethylene (LDPE) are different grades of polyethylene which are long chain polymer of ethylene^4^. LDPE is used in manufacturing disposable bags, grocery bags, food packaging etc. and contributes to 60% of total plastic production^5^. The accumulation of its waste led to environmental issues^6–8^. The conventional method of plastic disposal involves land filling, incineration, thermal degradation, etc.^7,9,10^ produces secondary pollutant/persistent organic pollutants causing further hazards to the environment^4,11^. Hence, safe remediation of plastic debris is very important for preserving the ecosystem. Therefore, the development of eco-friendly and sustainable plastic waste disposal methods are of interest in recent years. Bioremediation/Biodegradation is the only eco-friendly approach in plastic waste management for safe disposal of plastics^12–14^. Various researchers report the degradation of LDPE by bacteria, fungi, microalgae etc.^15–18^. These microorganisms grow and secrete extracellular enzymes and degrade the plastics and release oligomers/monomers^19,20^. Among the microbes, fungi comprise nearly 3% in the plastisphere and play a vital role in degradation of plastics^21^. Plastic degrading fungi (including *Aspergillus* spp., *Fusarium* spp., *Penicillium* spp.) have been detected from plastisphere by various researchers^3,22,23^. However, these fungi are reported to take longer duration for degradation. Therefore, this study focused on isolation of fungi which degrade LDPE in shorter duration.

## Materials and methods

### Preparation of LDPE powder and films

Polyethylene was purchased from Sigma-Aldrich (low density, melt index 25 g/10 min; 190 °C/2.16 kg, Cat. No. 428043). LDPE granules boiled with xylene and crushed to powder when it is warm^24^ and sterilized with 70% (v/v) ethanol. For films, commercially available LDPE bags were purchased from local markets (above 50 microns), cut into small pieces and sterilized with 70% ethanol and dried in a vacuum oven.

### Isolation and preliminary screening of LDPE degrading fungi

Plastic waste (single use plastic bags) from various garbage dumpsites and contaminated water bodies were collected in Chennai, Tamil Nadu, India (Supplementary Fig. S1) and stored in a sterile zip lock bag and brought to the laboratory. Fungi were isolated by spreading it on potato dextrose agar (PDA) medium. After 48 h of incubation, fungal colonies were selected and stained using lactophenol cotton blue and examined under a light microscope at 40X magnification. Isolated (pure) fungal cultures were maintained in PDA slants.

The individual culture was then inoculated on quarter strength potato dextrose broth (PDB) media supplemented with 0.5% (w/v) LDPE powder and incubated in an orbital shaker (100 rpm) at room temperature for 24 h. For control, medium without any fungus inoculation was used. The filtrate from both control and fungus inoculated was collected after centrifugation at 10000G for 10 min. The filtrate (50 µL) was introduced into a well of 0.5% (w/v) LDPE agarized petri plates (19 cm diameter) and incubated for 24 h at 37 °C. The plate was flooded with congo red stain, fixed with NaCl and the excess stain was removed. The halo zone of clearance was measured using a HiMedia zone measuring scale (in mm).

### Identification of fungi degrading LDPE

The fungus capable of degrading LDPE (showing distinct zone of clearance) was identified through morphological and molecular methods. Morphological identification of the fungus was carried out by Dr. Sanjay K. Singh at Agharkar Research Institute, Pune, India. Molecular identification was done by ITS amplification using ITS primers (Forward primer ITS1-[5ʹ-TCCGTAGGTGAACCTGCGG-3ʹ] and Reverse primer ITS4-[5ʹ-TCCTCCGCTTATTGATATGC3ʹ]). The genomic DNA was extracted by the method of^25^ with slight modifications. PCR reaction was carried out using the Eppendorf thermal cycler. The reaction condition was initial denaturation for 4 min at 94 °C followed by 30 cycles of denaturation at 94 °C for 1 min, annealing at 55 °C for 30 s and polymerization at 72 °C for 2 min. The amplified PCR product was electrophoresed on 1.2% (w/v) agarose gel, and the amplicon was purified and sequenced through outsourcing (Applied Biosystems, Bangalore). This was blasted with available sequence by BLASTN program. The sequence was submitted to NCBI GenBank and accession number was obtained (Accession Number OQ135202). Multiple sequence alignment was made using ClustalX2 and phylogenetic tree was constructed using neighbour joining tree method with Mega 11 software^26^.

### Biodegradation studies

#### Experimental design

The fungus *C. sphaerospermum* showing distinct zone of clearance on LDPE plate was selected for biodegradation of polyethylene films. A small piece of LDPE film (2 × 2 cm weighing ≈ 7 mg) was aseptically introduced into a quarter strength PDB medium and the pure fungal culture *C. sphaerospermum* (3 mm disc from 7 day old culture) was inoculated. Media containing a pre-weighed LDPE film (no fungal inoculation) served as a control. The experimental setup was maintained under controlled environmental conditions under static condition with optimum temperature of 35 °C for 7 days. After 7 days, the LDPE films were removed, washed and analysed for biodegradation. The experiment was done in triplicate.

The culture filtrate collected after centrifugation at 10,000 G for 10 min was used to analyse the degradation products through GC–MS analysis. The filtrate was also used to quantify laccase (Lac), Manganese peroxidase (MnP) and Lignin peroxidase (LiP). Protein content was evaluated according to^27^.

Laccase was assayed following the procedure of Kalra et al.^28^. The reaction mixture consists of one mL of culture filtrate, 1 mL of 2 mM guaiacol and 3 mL of 10 mM sodium acetate buffer (pH 4.6), incubated at 30 °C for 15 min and read at 450 nm. Lac was expressed in Units, where one unit is the amount of enzyme required to oxidize 1 µmol of guaiacol in one minute.

MnP was assayed following the procedure of Paszcynski et al.^29^. The reaction mixture consists of 100 µL of culture filtrate, 200 µL of 0.5 M sodium tartarate buffer (pH 5.0) to which 1 mL of 1 mM guaiacol, 100 µL of 1 mM MnSO_4_ and 400 µL of distilled water. The reaction was initiated by adding 100 µL of 1 mM H_2_O_2_ and the absorbance was read at 465 nm. One unit of MnP is the amount of enzyme required to oxidize 1 µmol of guaiacol in one minute.

For LiP assay, the assay mixture consisted of 1 mL of 125 mM sodium tartarate buffer (pH 3), 500 µL of 10 mM veratryl alcohol and 500 µL of culture filtrate. The reaction was initiated by the addition of 500 µL of 200 mM H_2_O_2_ and absorbance was read at 310 nm^30^. One unit of LiP is the enzyme required for oxidation of 1 µmol of vertaryl alcohol to veratrylaldehyde in one minute.

### Weight loss measurement

The LDPE strip was carefully removed from the flask and rinsed well with 30% (w/v) SDS for removal of adherent mycelia, followed by 70% (v/v) ethanol wash, sterile distilled water and dried in vacuum oven at 35 °C. The LDPE strip was weighed using an electronic weighing balance. The percentage weight loss was calculated using the following formula^13^

$$\text{Percentage we}\text{ight loss}= \frac{\mathrm{Initial\; weight}-\mathrm{Final \;Weight}}{{\text{Initial}}\;\mathrm{ Weight}} \times 100$$

With the acquired data, the reduction rate constant (*K*) of LDPE was calculated using the following equation:

$$ {\text{Reduction}}\;{\text{rate}}\;{\text{constant}}\left( {\text{K}} \right) =  - \frac{1}{{\text{t}}}\left( {{\text{ln}}\frac{{{\text{Final}}\;{\text{weight}}}}{{{\text{Initial}}\;{\text{weight}}}}} \right) $$

where t denotes the degradation time in days. The result (K) was used to determine the half-life (t_1/2_) of the treated LDPE using the following formula^31^:

$${t}_{1/2}=0.693/K$$

### Microscopic analysis

#### Optical microscope

The LDPE strip was observed under Leica ATC 2000 at 10X magnification to assess the adsorption and colonization of fungus on LDPE strip.

### SEM analysis

The changes in surface morphology of the fungus treated LDPE strip was analysed by comparing the results of untreated control LDPE strip through SEM (TESCAN VEGA3) at 30 kV. The LDPE strip was washed with 30% SDS (w/v) to remove any microbial debris, vacuum dried and coated with gold sputter.

### AFM analysis

The surface erosion/modification was also evaluated by comparing both control and *C. sphaerospermum* treated LDPE strip using Atomic Force Microscopic analysis (Park Systems XE-70). The images were obtained with a scan speed of 1.0 Hz.

### FTIR analysis

The changes in polymer bonds of *C. sphaerospermum* treated LDPE strip was analyzed using a FTIR spectrophotometer (BRUKER-ALPHA Platinum ATR-IR) by KBr pellet method. The pellets were scanned in the region of 500 to 4000 cm^−1^. The carbonyl index (CI) was assessed for various bonds like ester bond (ECI), vinyl bond (VI), keto carbonyl bond (KCI) and internal double bond (IDI) to measure the degree of biodegradation with the following formula^14^:

$$ ECI = A1740/A1465; $$

$$ VI = A1650/A1465; $$

$$ KCI = A1715/A1465; $$

$$ IDI = A1908/A1465 $$

The crystallinity percentage of the LDPE strip was determined using the following formula^32^:

$$ {\text{Crystallinity }}\left( \% \right) = \left( {\frac{{1 - \left( {\frac{{({\text{A}}1474/{\text{A}}1464}}{1.233}} \right)}}{{1 + \left( {\frac{{{\text{A}}1474}}{{{\text{A}}1464}}} \right)}}} \right) \times 100 $$

From these results the reduction percentage was calculated:

$$ {\text{Reduction }}\left( {\text{\% }} \right) = { }\frac{{{\text{C}} - {\text{T}}}}{{\text{C}}} \times 100 $$

where C is the crystallinity % in control untreated LDPE film and T is the crystallinity % in *C. sphaerospermum* treated LDPE film.

### GPC analysis

Molecular weight differences between fungus treated polyethylene and untreated control polyethylene was measured by high temperature gel-permeation chromatography (HT-GPC) at a temperature of 150 °C with a liquid phase containing tetrachlorobenzene (TCB) using viscometric detector.

### GC–MS analysis

The culture filtrate (both control and *C. sphaerospermum* treated) was extracted with ethanol and used to identify the degradation products of LDPE, using GC–MS analysis (Shimadzu QP2020, Japan). One microliter of sample was injected through AOC-20i auto injector. The oven temperature was held at 50 °C, increased to 280 °C at a rate of 6 °C/min. The Ion source was maintained at a temperature of 200 °C at a threshold of 1000. A whole scan range from 50 to 500 m/z with a scan time of 0.30 s was setup.

### Statistical analysis

All the experiments were carried out in triplicates (n = 3) and the results are presented in mean value with standard deviation. All statistical analysis was performed with GraphPad Prism 7.0 software.

## Results and discussion

### Preliminary screening and identification of LDPE degrading fungal isolates

**Figure 1:**
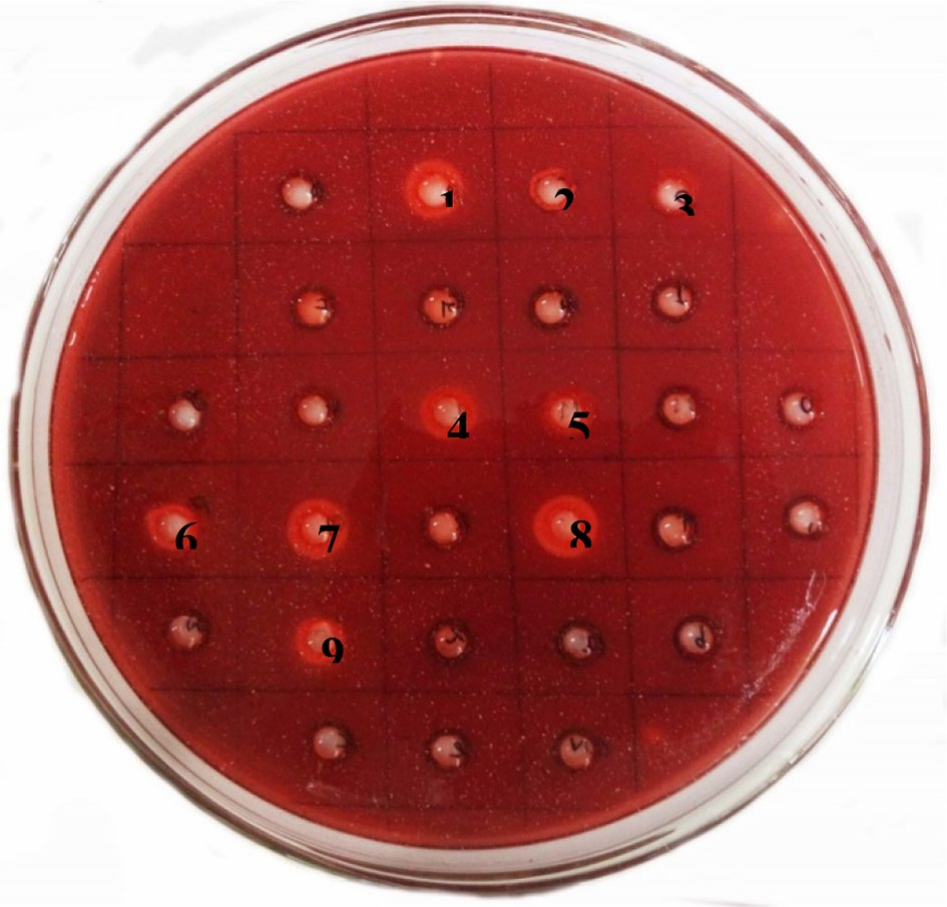
Culture filtrate of isolated fungi showing halo zone of clearance in LDPE agarized plate. [1. 21S1B, 2. 21S1C, 3. 21S1D, 4. 21S3L, 5. 21S3M, 6. 21S3P, 7. 21S3Q, 8. 21S4S, 9. 21S4Y].

A total of 33 fungi were isolated (Table 1) from the plastisphere and out of which 28 fungal species were found to be capable of utilizing LDPE as a carbon source. The culture filtrate of these fungi showed halo zone of clearance in agarized LDPE media (Fig. 1). Maximum clearance zone of 16 mm was observed in fungus 21S4S followed by 21S1B and 21S3Q with 14 mm clearance zone at 24 h. Therefore, the fungus 21S4S was used in this study.

**Table 1:** Details of sampling site with location coordinates and the fungi isolated from the respective plastisphere.

Locality	Type	Coordinates	Sample no.	No. of fungi isolated
Palliagraharam waste dump	Garbage dump marsh banks	12.9349ʹ N; 80.2137ʹ E	21S1A, 21S1B, 21S1C, 21S1D, 21S1E, 21S1F, 21S1G	7
Perungudi dump yard	Municipal waste dump site	12.9547ʹ N; 80.2315ʹ E	21S2H, 21S2I, 21S2J, 21S2K	4
Madipakkam lake	Fresh water source	12.9610ʹ N; 80.1923ʹ E	21S3L, 21S3M, 21S3N, 21S3O, 21S3P, 21S3Q, 21S3R	7
Velachery	Marsh water region	12.9547ʹ N; 80.2315ʹ E	**21S4S**, 21S4T, 21S4U, 21S4V, 21S4W, 21S4X, 21S4Y, 21S4Z, 21S4a, 21S4b, 21S4c, 21S4d, 21S4e, 21S4f, 21S4g	15

Based on the morphological characters, the fungus (21S4S) was identified as *Cladosporium sphaerospermum* (culture code 2054-S). The molecular identification also revealed the same with 90.43% similarity (Accession number OQ135202). Multiple sequence alignment was shown in Supplementary Fig. S2. The phylogenetic tree constructed showed a major clade with *C. sphaerospermum* and showed the phylogenetic relationship between other species of *Cladosporium* (Supplementary Fig. S3). The outgroup in the phylogentic tree was *Amanita muscaria*, *Chaetomium globosum* and *C. thermophilum*.

### Biodegradation

#### Weight loss measurements

Polymer deterioration is measured by weight difference, SEM etc.^22,33^. The analytical method of weight loss measurement was used initially to evaluate the biodegradation process after 7 days. Significant weight loss (%) of 15.12% was observed in *C. sphaerospermum* treated LDPE film whereas there was no change in weight was observed in control. El-Sayed et al.^2^ reported 3.8%, 2.26% weight loss by *Aspergillus carbonarius* and *A. fumigatus* respectively on 30 days whereas mixed culture showed a 5.85% weight loss under the same condition. Nearly 35% and 38% weight loss in 90 days by *A. clavatus* and *Penicillium citrinuum* respectively was reported^34,35^. Though weight loss was high in these cases, it takes nearly 90 days whereas *C. sphaerospermum* used in this study showed weight loss in a week time. The weight loss in LDPE films treated with *C. sphaerospermum* might be due to consumption of LDPE as a carbon source which is in accordance with previous reports^1,36^. The reduction rate (k) of LDPE by *C. sphaerospermum* was found to be 0.0224/day and the half-life (t_1/2_) was 30.93 days. Roughly 31 days might be required to reduce the PE strip to half the weight. This was the first report on the degradation of LDPE by *C. sphaerospermum* in a very short duration. These results indicate that during the nutrient depleted conditions, *C. sphaerospermum* might utilize LDPE and degrade it. Increased rate of biodegradation by consortium of *Aspergillus* sp. results in weight loss was reported by Dsouza et al.^19^. Poitin et al.^37^ reported that *C. sphaerospermum* isolated from aged gas plant efficiently degraded polyaromatic hydrocarbons. There are reports that bacteria as well as few microalgae degrade LDPE but the half-life time to degrade LDPE by these organisms were very high^14,18,38^. This showed that the fungus is more potent than other microorganisms in degradation of LDPE. LDPE degradation by *Trichoderma viride*, *Paecilomyces varioti*, *Aureobasidium pullulans* were also reported^3,4^. Gong et al.^39^ reported that heat treatment followed by *Cephalosporium sp.* treatment result in weight loss of LDPE in 30 days. The fungus *Neopestalotiopsis phangngaensis* degrade LDPE after 90 days incubation was reported by^40^.

### Optical microscopy

The growth of *C. sphaerospermum* on the LDPE strip was observed under the microscope (Supplementary Fig. S4).

### SEM analysis

**Figure 2:**
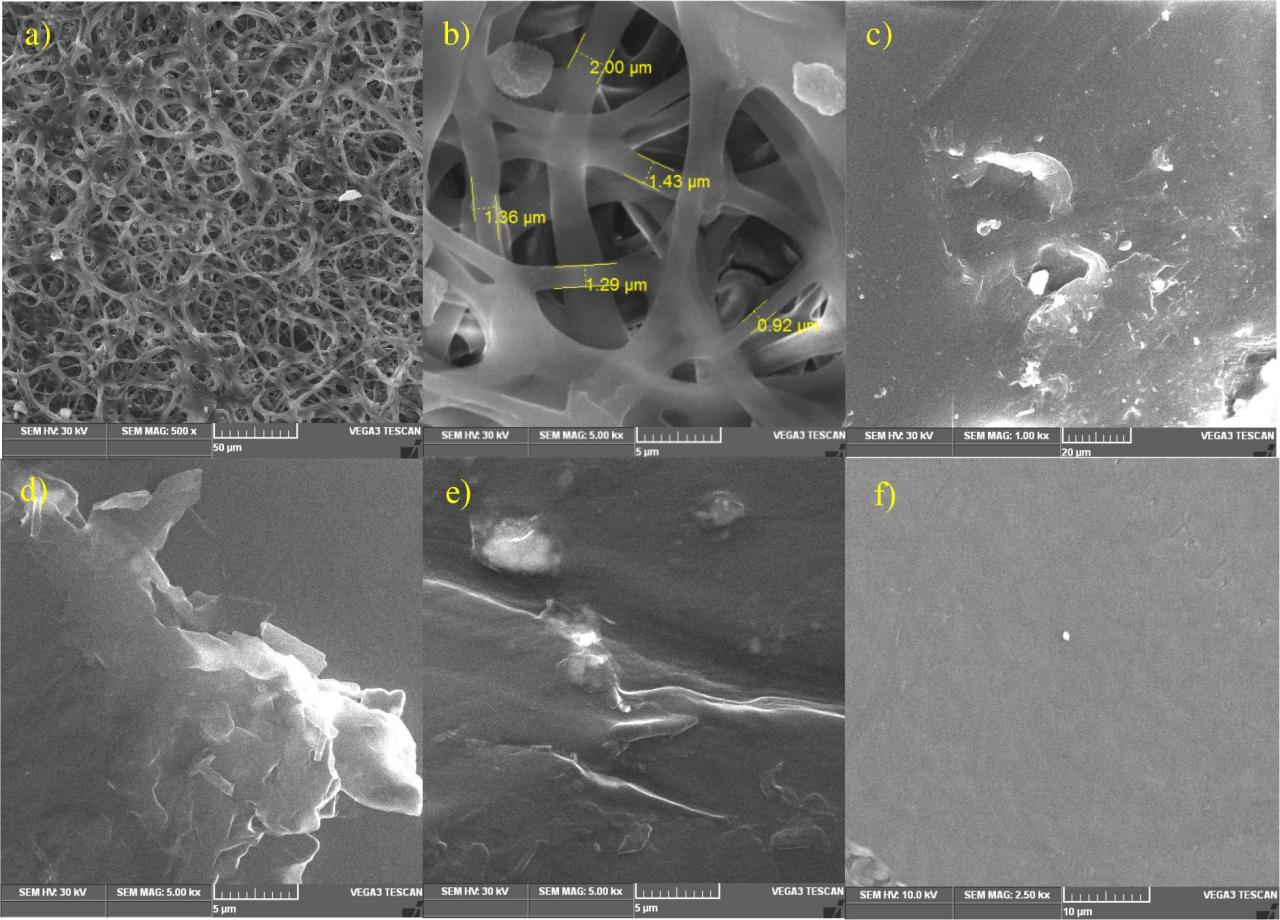
SEM image of *C. sphaerospermum* treated LDPE film. Colonization of mycelia on the surface (**a**, **b**); pits, cracks and aberrations in treated LDPE film (**c**, **d**, **e**); Control untreated LDPE film (f)

The SEM micrographs of LDPE film treated with *C. sphaerospermum* and untreated control was shown in Fig. 2. Mycelial adherence and thick biofilm formation was observed on the LDPE strip even after several washes with SDS (Fig. 2a,b). Similar type of results of fungal adhesion and penetration was reported by^2^. The LDPE strip after a thorough wash with SDS processed for SEM showed surface changes in *C. sphaerospermum* treated LDPE. SEM image of treated LDPE showed worn and deteriorated surface in the form of pits, cracks and cavities (Fig. 2c–e) whereas the surface of the untreated control LDPE strip had a smooth surface with no defects (Fig. 2f). Hence, this might be due to the penetration of the fungus by utilizing the carbon chain of LDPE and contributes to biodegradation. This result is in accordance with previous reports of LDPE degradation by *Aspergillus* sp.^1,19^. It has been reported that fungal penetration resulted in physical weakening and disintegration of LDPE strip^1^. Sanyasi et al.^18^ reported the appearance of pits, grooves, and corrosion on LDPE strip treated with microalgae due to adhesion molecules or by lignin degrading enzymes.

### AFM analysis

**Figure 3:**
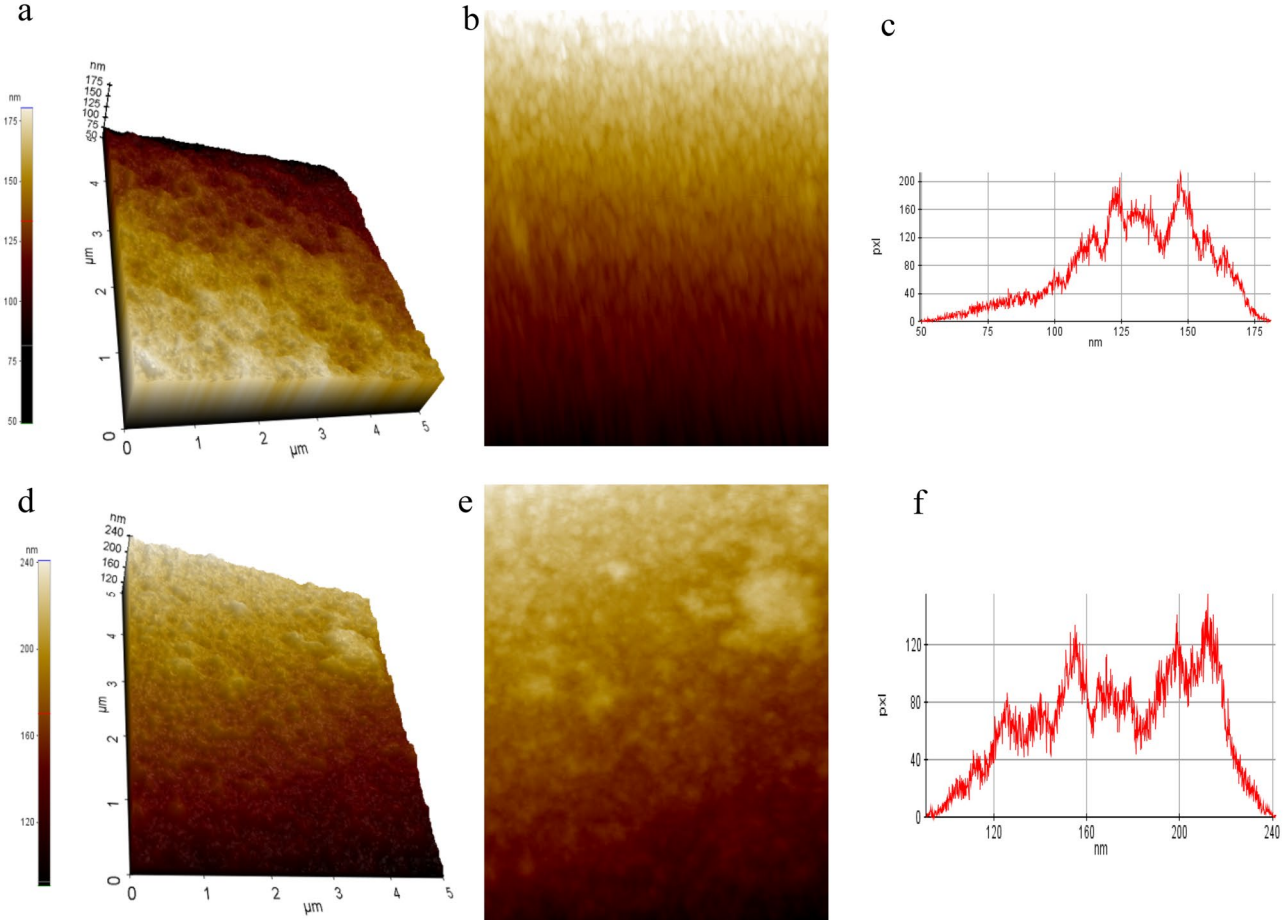
AFM topography image of Control (**a**) and *C. sphaerospermum* treated (**e**) LDPE films. Grain images of LDPE films, control (**b**) showing even distribution of grain and *C. sphaerospermum* (**d**) treated with uneven distribution of grains. The height distribution histogram of control (**c**) with maximum height in the range of 125 nm to 150 nm whereas *C. sphaerospermum* treated (**f**) LDPE films showing heights at 120 to 220 nm evidencing the increased surface roughness due to biological activity.

**Figure 4:**
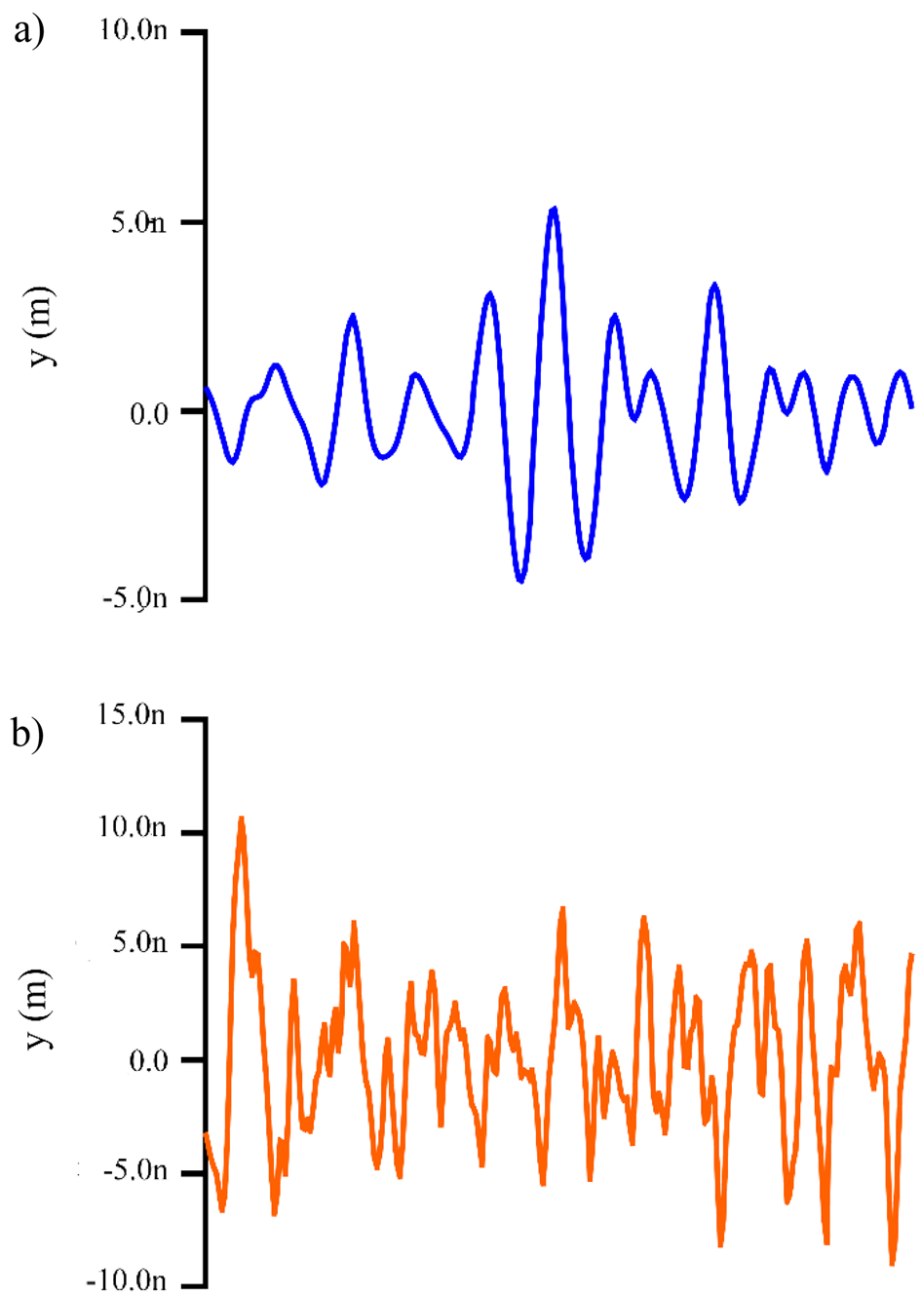
Surface roughness of control (**a**) and *C. sphaerospermum* treated (**b**) LDPE film.

To further confirm the results obtained from SEM analysis, AFM analysis was carried out both in control (fungus untreated LDPE) and treated (*C. sphaerospermum* treated LDPE) sample. The 3D images of *C. sphaerospermum* treated LDPE film showed an increase in roughness and surface erosion whereas in control strip no change was observed (Figs. 3 and 4).

**Figure 5:**
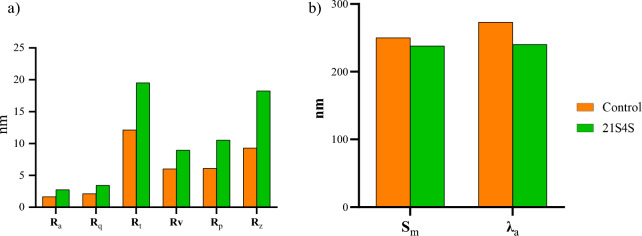
The surface parameters of control and *C. sphaerospermum* treated LDPE film. Amplitude (**a**) pertaining to roughness average (R_a_), root mean square roughness (R_q_), maximum height of roughness (R_t_), maximum roughness valley depth (R_v_), maximum roughness peak height (R_p_), average maximum height of the profile (R_z_); spatial parameters (**b**)—mean spacing profile irregularities (S_m_) and average wavelength of the profile (λ_a_).

Amplitude and spatial parameters such as roughness average (R_a_), root mean square roughness (R_q_), maximum height of roughness (R_t_), maximum roughness valley depth (R_v_), maximum roughness peak height (R_p_), average maximum height of the profile (R_z_), mean spacing profile irregularities (S_m_) and the average wavelength of the profile (λ_a_) were determined. Roughness average (R_a_), roughness peak height (R_p_) and roughness valley depth (R_v_) are the important factors in determining the biodeterioration of LDPE sheet surface^18^. The increase in roughness peak height and valley depth in *C. sphaerospermum* treated LDPE strip when compared to fungus untreated control LDPE film (Fig. 5a) clearly indicates the surface erosion in *C. sphaerospermum* treated LDPE strip. As a result, the average roughness increased in *C. sphaerospermum* treated LDPE strip (Fig. 5a). The increase in peak height (1056 nm) and valley depth (8.971 nm) in *C. sphaerospermum* treated samples in comparison to control (6.116 nm and 6.028 nm respectively) clearly indicates the surface erosion. As a result, the average roughness increased in C*. sphaerospermum* treated LDPE strip in comparison to control. There was a decrease in S_m_ and λ_a_ in *C. sphaerospermum* treated LDPE strip was recorded (Fig. 5b) which supports the biodegradation of LDPE film by the fungus. All the parameters evidenced the surface changes in the LDPE strip due to *C. sphaerospermum* activity. Ojha et al.^4^ reported the increase in roughness due to formation of grooves and cracks in the LDPE film treated with *P. oxalicum* and *P. chrysogenum* for 90 days. Fungal enzymes act on the surface of the LDPE strip which corrodes the surface molecules subsequently increasing the surface roughness.

### Lignolytic enzymes

**Figure 6:**
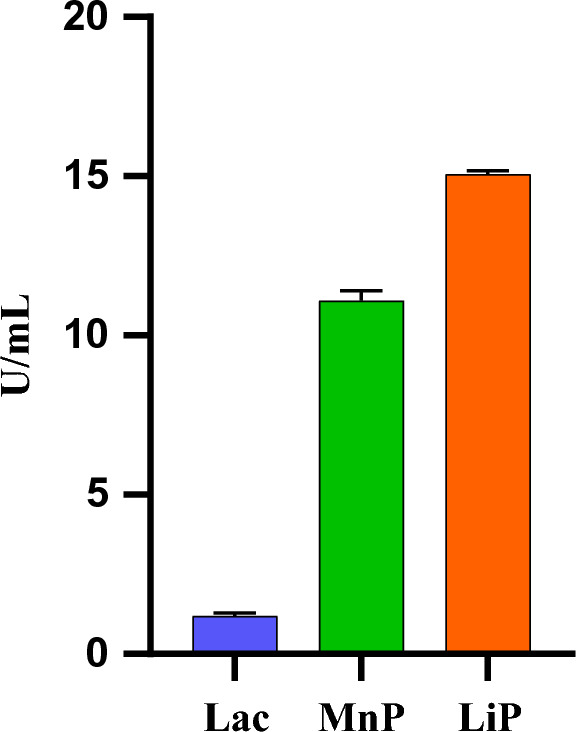
Extracellular lignolytic enzymes of *C. sphaerospermum* grown in LDPE film after 7 days. (Lac: Laccase, MnP: Manganese Peroxidase, LiP: Lignin Peroxidase).

The assimilation of larger complexes into a cell can be facilitated by production of complex enzymes, particularly extracellular enzymes that could bind to the hydrophobic surfaces and acts in cleavage of long chains^2^. Lignolytic enzymes play a key role as natural surface activators, by oxidation it creates surface morphological changes that could result in increased surface compatibility^41^. Polyethylene degradation by microbes facilitated through Lac, MnP and LiP has been reported^38,41–43^. Hence, the major lignolytic enzymes viz., Lac, MnP and LiP were quantified in the culture filtrate of *C. sphaerospermum* grown in LDPE medium on the 7th day. The extracellular protein content was quantified to be 20.67 ± 0.29 µg/ml. Both the peroxidases (MnP and Lip) production was high in LDPE film incubated with *C. sphaerospermum* whereas Lac was produced in minimal amount (Fig. 6). These results are in accordance with previous reports that adherence of fungal mycelia to LDPE film is followed by enzyme production and results in surface aberrations and erosion^44,45^. Lac, MnP and LiP produced by microbes involved in biodegradation of PE^46^ by enhancing the hydrophilicity of PE, allows the microorganisms to attach to PE^45,47^ and degrade the polymers into small oligomers, dimmers and monomers^40^. Weight loss in LDPE by microalgae due to lignolytic enzymes was reported by^48^. Dsouza et al.^19^ reported that the weight loss in LDPE film treated with *Aspergillus* sp. is due to the action of enzymes that break the lignolytic polymers into monomers and oligomers which could be directly utilized by the organism. In this study, we have observed weight loss, fungal adherence, pits and cracks in SEM analysis in LDPE strip treated with *C. sphaerospermum* and production of extracellular lignolytic enzymes by *C. sphaerospermum* grown in LDPE containing medium. It might be possible that the lignolytic enzymes produced by *C. sphaerospermum* might break the LDPE polymer into simple oligomer/monomer and degrade it.

### FTIR analysis

**Figure 7:**
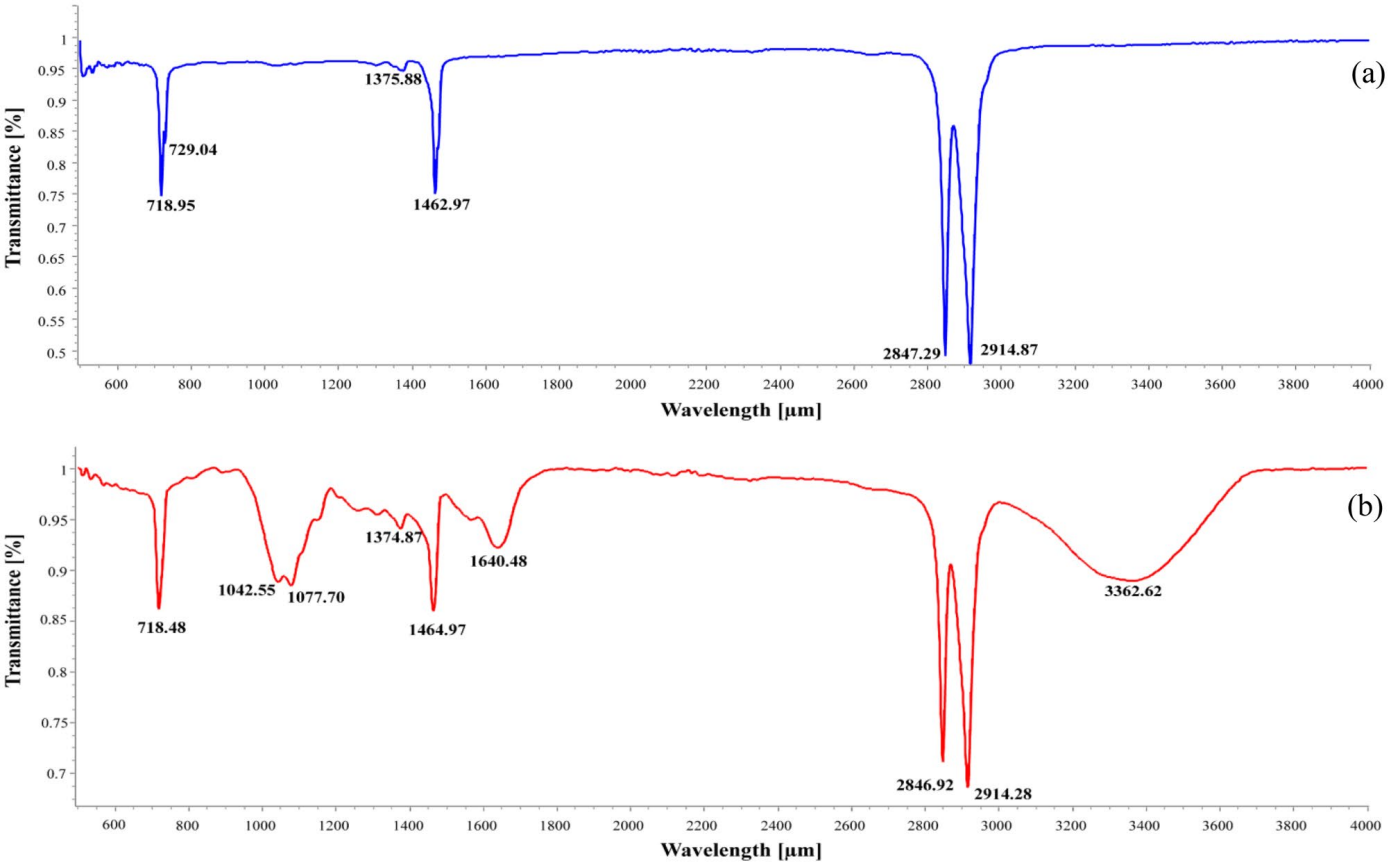
FTIR spectra of control (**a**) and *C. sphaerospermum* treated (**b**) LDPE film.

Assimilation of plastics by the fungi can be measured by FTIR^33^. Changes in the structural and functional group can be determined through FTIR which could possibly explain the weight loss in the LDPE film. The peaks at 2914 cm^−1^, 2847 cm^−1^ corresponds to CH_2_ asymmetric and symmetric stretches respectively. The bending deformation at 1462 cm^−1^ and peaks at 729 cm^−1^ and 718 cm^−1^ corresponds to rocking deformation which is characteristics of polyethylene (Fig. 7a). The LDPE strip treated with *C. sphaerospermum* showed an intensity drop in wavelength 2914 cm^−1^ region (Fig. 7b). There was a shift in peak to 2846.92 cm^−1^ and 1464.97 cm^−1^ in comparison to control that correlates to CH_2_ symmetric deformation. Alike deformation caused by *Cephalosporium* sp. was reported by^49^. A strong, broad peak at 3362.62 cm^−1^ that corresponds to hydroxyl group and a small stretch at 1042.55 cm^−1^ indicating C–O group (anhydride) was observed in the treated LDPE film. Another peak at 1077.70 cm^−1^ which corresponds to primary alcohol was also detected in LDPE treated with *C. sphaerospermum* (Fig. 7b). FTIR spectra showed appearance of new functional groups assigned to hydrocarbon degradation confirming the role enzymes. The peaks at 729 cm^−1^ and 718 cm^−1^ corresponds to CH_2_ rocking deformation which is in accord with previous report^50^. These shifts in the peaks, reduction in peak intensity and formation of new peaks can be put forth as a result of biodegradation.

**Figure 8:**
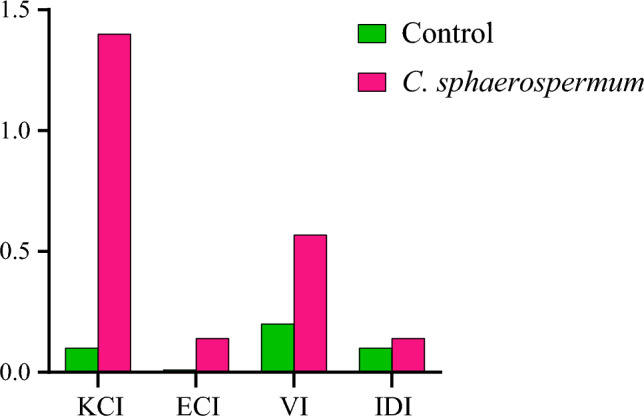
The carbonyl index (CI) was assessed for various bonds like ester bond (ECI), vinyl bond (VI), keto carbonyl bond (KCI) and internal double bond to measure the degree of biodegradation in* C. sphaerospermum* treated LDPE strip.

Biodegradation of polyethylene involves oxidation of polymer chains as a first step and results in production of carbonyl groups^14^. Therefore, carbonyl index was used to measure the degree of biodegradation^51^. For this, FTIR indices of ester, keto, vinyl and internal double bonds were used to evaluate LDPE biodegradation. An increase in the carboxyl index (KCI, ECI, VI and IDI) was observed in the *C. sphaerospermum* treated LDPE (Fig. 8) which further confirms that *C. sphaerospermum* play role in biodegradation of LDPE. These results are in accord with^4^. As biodegradation progresses, these oxidized polymers are consumed by the microbes^52^. During biodegradation, various enzymes catalyse the chemical reactions viz., oxidation, reduction, esterification etc. Among them, keto and ester carbonyl bonds were reported to be the major products during oxidoreductase reaction^53^.

The crystallinity percentage of fungus untreated control LDPE was 31.6% whereas *C. sphaerospermum* treated LDPE was 27.3% which denotes the deterioration of LDPE by *C. sphaerospermum*. Gowthami et al.^14^, reported that when deterioration progresses, the crystallinity percentage reduces. In our study, the crystallinity reduction of 13.5% was observed. A maximum crystallinity reduction of 8.5% was reported in LDPE treated with *Bacillus spericus*^54^. LDPE biodegradation is associated with change in physical and chemical properties^19^. In this study also we found the loss of weight, change in surface functional groups etc.

### GPC analysis

GPC analysis showed that *C. sphaerospermum* treated polyethylene reduced the Mn and Mw when compared to untreated control (Table 2). The molecular weight of fungus treated LDPE decrease and the dispersion index was increased which indicates the depolymerisation of LDPE by *C. sphaerospermum.* This *might* be due to the lignolytic enzymes produced by *C. sphaerospermum.* The results of the present study are in accord with the previous reports on polyethylene degradation by microbes^55,56^.

**Table 2:** GPC analysis of LDPE film after ten days exposure with *C. sphaerospermum.*

Sample	M_n_	M_w_	M_n_/M_w_
Control (Initial)	18,630	76,562	4.10
Treated	16,830	74,490	4.42

### Gas chromatography and mass spectrometric analysis

**Figure 9:**
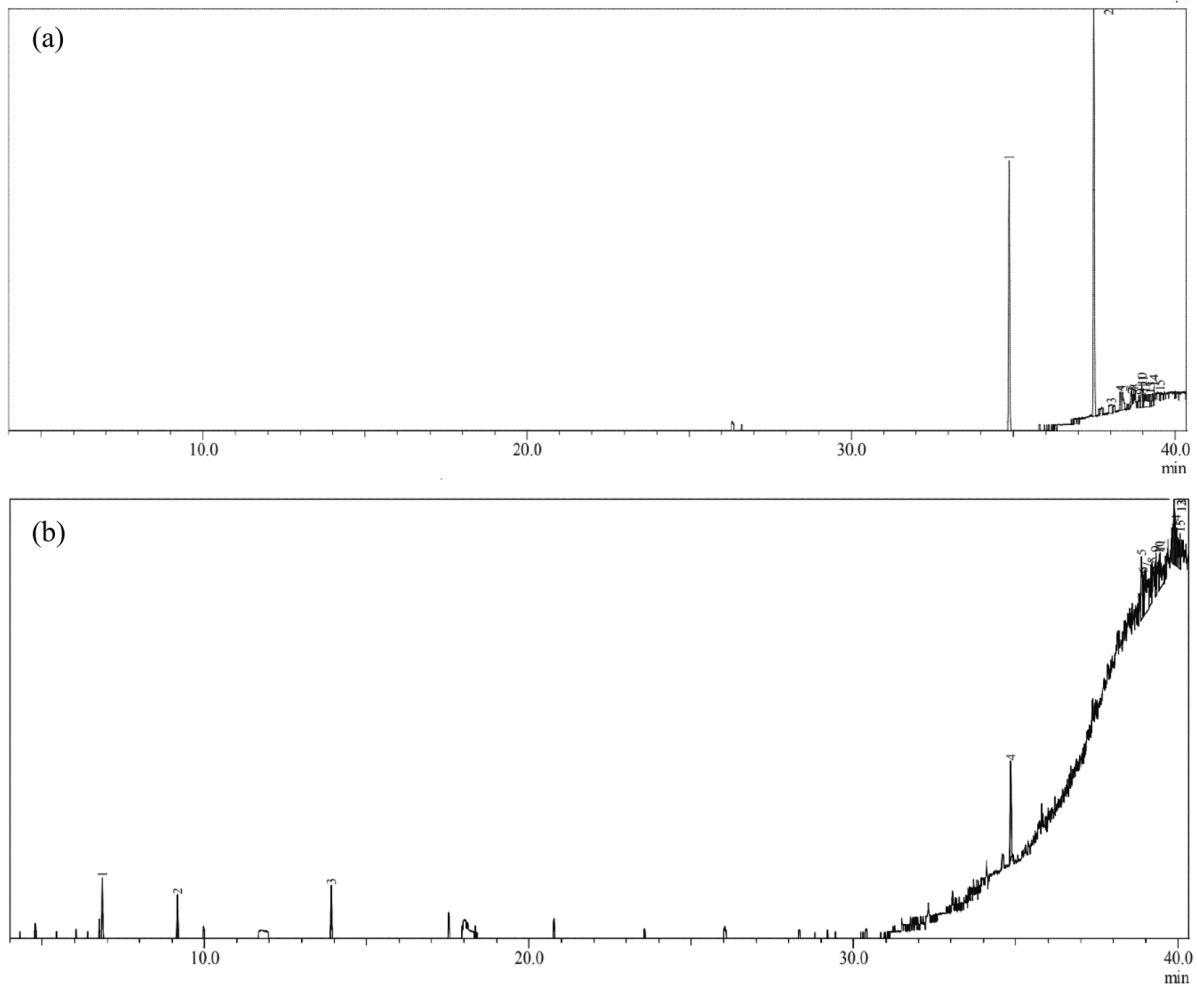
GC–MS spectra of control (**a**) and *C. sphaerospermum* (**b**) treated LDPE film.

GC–MS analysis of extract of *C. sphaerospermum* grown in LDPE showed significant difference in components when compared to the untreated control (Fig. 9). In control, the presence of benzene dicarboxylic acid (RT 34.825 and RT 37.482) was observed. Due to the degradation of LDPE by *C. sphaerospermum* several short chains of alkanes, carboxylic acids and other molecules were identified in the extract. Several short chained carbon compounds such as dodecane (RT 6.653), hexasiloxane (RT 38.869) and silane (RT 39.183) were identified in the extract of *C. sphaerospermum* grown on LDPE. Alcohols and carboxylic acids such as 1-Eicosanol (RT 40.063) and dihydroxy benzoic acid (39.945) and succinic acid (RT 39.413) were also observed in the extract of *C. sphaerospermum* grown in LDPE (Table 3). A large number of short-chained hydrocarbons were present in the extract of *C. sphaerospermum* grown in LDPE which might be due to biodegradation. Awasthi et al.^57^ reported the presence of alkanes and carboxylic acids as a result of LDPE degradation by *Rhizopus oryzae*. GC–MS analysis of PE showed release of fatty acids due to esterase activity was reported by Khandare et al.^58^. They also reported that extracellular enzymes like esterase, lignin peroxidase, laccase etc. produced by microorganisms are responsible for degrading plastic. Similar type of results was also reported by microbial degradation^2,18^. Pretreatment of plastics prior to biodegradation was reported to be efficient by Rad et al.^59^.

**Table 3:** GC–MS analysis of *C. sphaerospermum* treated LDPE film.

Peak	Retention time	Area %	Name
1	6.853	5.28	Dodecane, 1,1-Difluoro-
2	9.173	4.24	Dodecane, 1,1-Difluoro-
3	13.911	5.94	2,3,4,4-tetrapropyl-1-(trimethylsilyl)-1-(trimethylsilyloxy)-1,3-diaza-2,4-diborabutane
4	34.84	12.59	1,2-Benzenedicarboxylic acid, Dioctyl ester
5	38.869	9.83	Hexasiloxane, tetradecamethyl-
6	38.92	3.3	Carbamazepine-10,11-dihydro-10-ol,2TMS derivative
7	38.99	13.79	4-Oxatricyclo[5.1.0.0(1,3)]octan-5-one
8	39.183	8.25	Silane, diethylisohexyloxy(pentafluorobenzyloxy)-
9	39.31	3.46	2-Cyclohexyl-2-hydroxyethanamide
10	39.413	2.26	Succinic acid, 2,2,3,3,4,4,4-heptafluorobutyl 2-methylhex-3-yl ester
11	39.453	8.39	2-Furancarboxamide, N-(4-bromophenyl)-
12	39.865	7.47	(1.alpha.,4.alpha.,4a.alpha.,7.alpha.,8a.beta.)-4-[(tert-Butyldimethylsilyl)oxy]decahydro-1,4a,8,8-tetramethyl-1,7-naphthalene
13	39.89	6.93	5-Iminopyrrolidine-2-carbonitrile
14	39.945	4.05	2,3-Dihydroxybenzoic acid, 3TMS derivative
15	40.063	4.24	1-Eicosanol, TBDMS derivative

## Conclusion

The present study isolated and identified *C. sphaerospermum,* a potential fungus from the plastisphere which degrades the LDPE efficiently. Biodegradation of LDPE film was characterized by weight loss, FTIR, SEM, AFM, GPC and GC–MS analyses. A weight loss of 15.12% was observed in *C. sphaerospermum* treated LDPE film in 7 days. Microscopic analysis showed the adherence of fungal mycelium on LDPE film. SEM analysis revealed the changes on the surface of treated LDPE film in the form of cracks, pits and cavities. This was confirmed by AFM analysis which showed increased roughness and surface erosion in treated LDPE film. The *C. sphaerospermum* secretes extracellular lignolytic enzymes viz., MnP and LiP when grown in LDPE containing medium. The changes observed in SEM might be due to the activity of these extracellular enzymes produced by *C. sphaerospermum*. FTIR analysis indicated a change in surface functional groups in LDPE treated with the fungus. In GC–MS analysis several short chains of alkanes, carboxylic acids and other small molecules were identified in the extract of *C. sphaerospermum* grown in LDPE. This further confirms the significant degradation of LDPE by *C. sphaerospermum.* The results of this study demonstrated that *C. sphaerospermum* can be used as a potential microbe to degrade the LDPE in shorter duration.

### Supplementary Information

Supplementary Figures.

## Data Availability

Correspondence and requests for materials should be addressed to M.S.
