# Supplementary material for: Mycodegradation of low-density polyethylene by Cladosporium sphaerospermum, isolated from platisphere
Source: Sci Rep. 2024 Apr 9;14:8351. doi: 10.1038/s41598-024-59032-4 (PMC11004025; doi:10.1038/s41598-024-59032-4)
Supplement: Supplementary file 1 — Supplementary Figures. [file 41598_2024_59032_MOESM1_ESM.docx]

**Mycodegradation of Low-density Polyethylene by *Cladosporium sphaerospermum*, isolated from platisphere**

**M. Sathiyabama***^a^, R. V. Boomija^a^, T. Sathiyamoorthy^b^, N. Mathivanan^b^, R. Balaji^b^

^a^ Department of Botany, Bharathidasan University, Tiruchirappalli 620 024, Tamil Nadu, India.

^b^ CAS in Botany, University of Madras, Chennai 600025, Tamil Nadu, India.


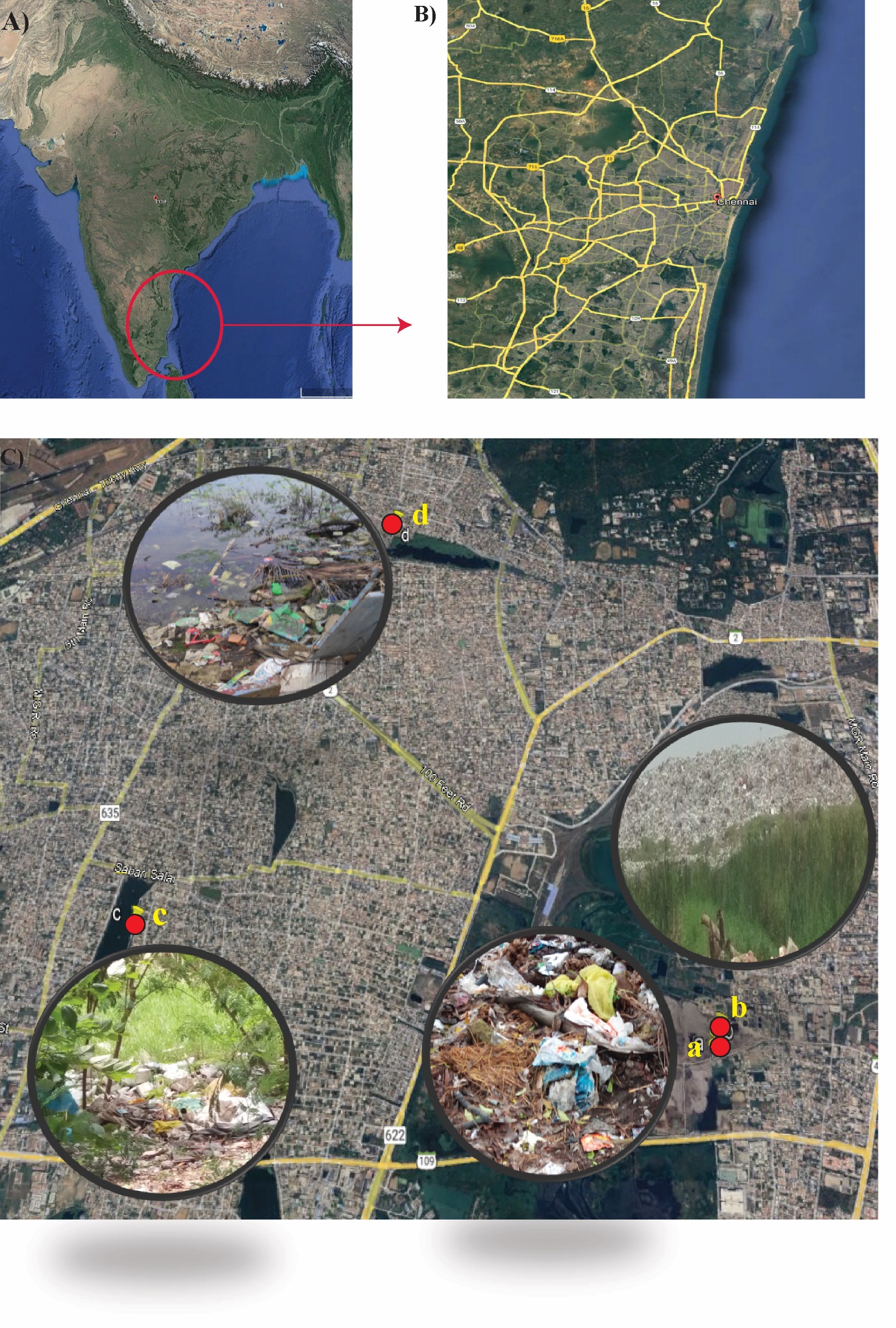


**Supplementary Figure S1:** A view of Indian subcontinent with sampling site specified (A); Chennai city, the major area of sampling was zoomed in (B); Places where rugged worn out plastic wastes were collected (C) were pinned along with their location images – Palliagraharam waste dump (a), Perungudi (b), Madipakkam lake (c) and Velachery (d). These maps were created using the software Google EarthPro 7.3.6.9345 (64 bit) (<https://www.google.com/intl/en_in/earth/about/versions/download-thank-you/?usagestats=1>)


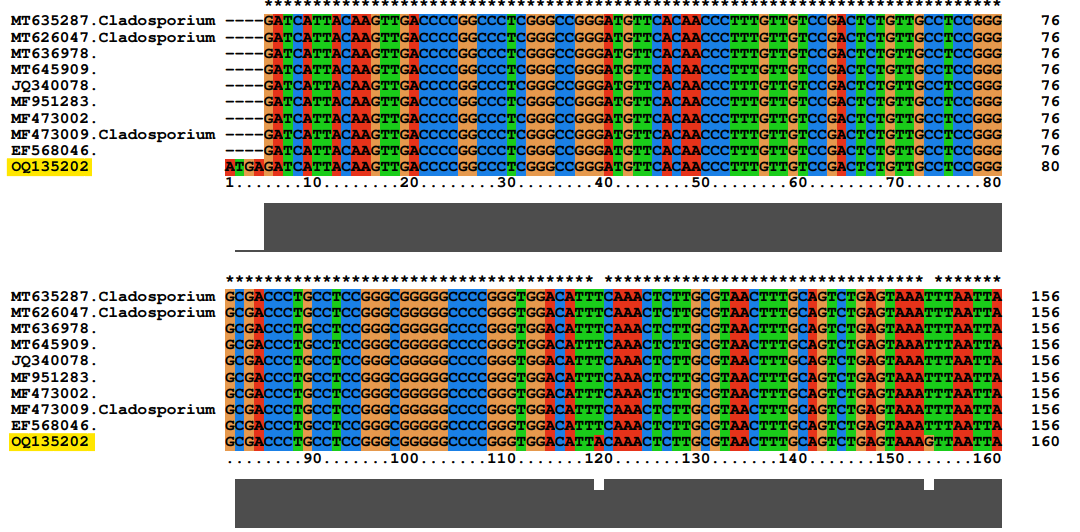

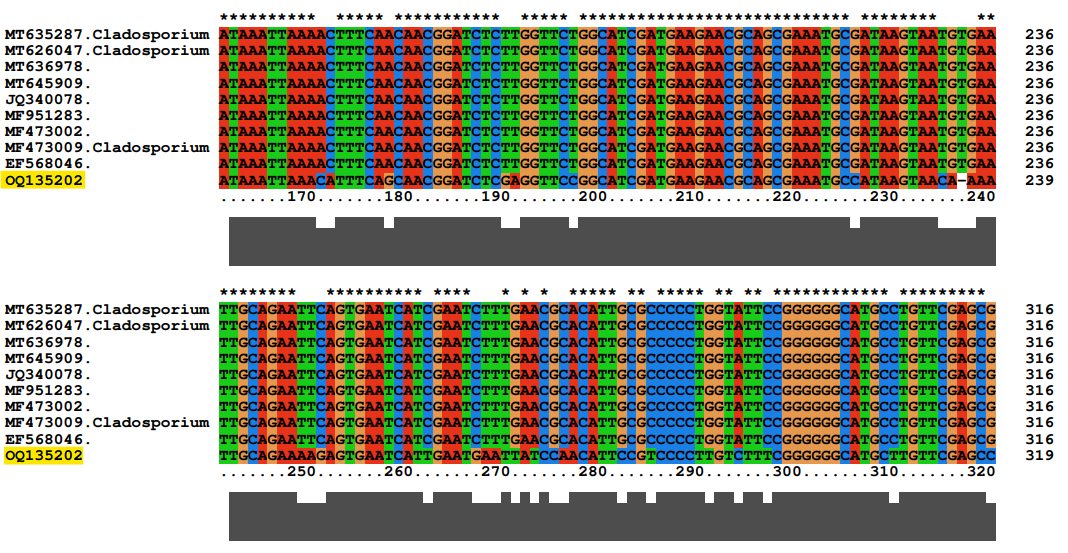

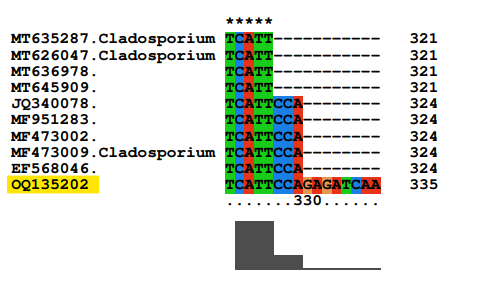


**Supplementary Figure S2.**  Multiple sequence alignment of C. sphaerospermum (Acc. No.: OQ135202)


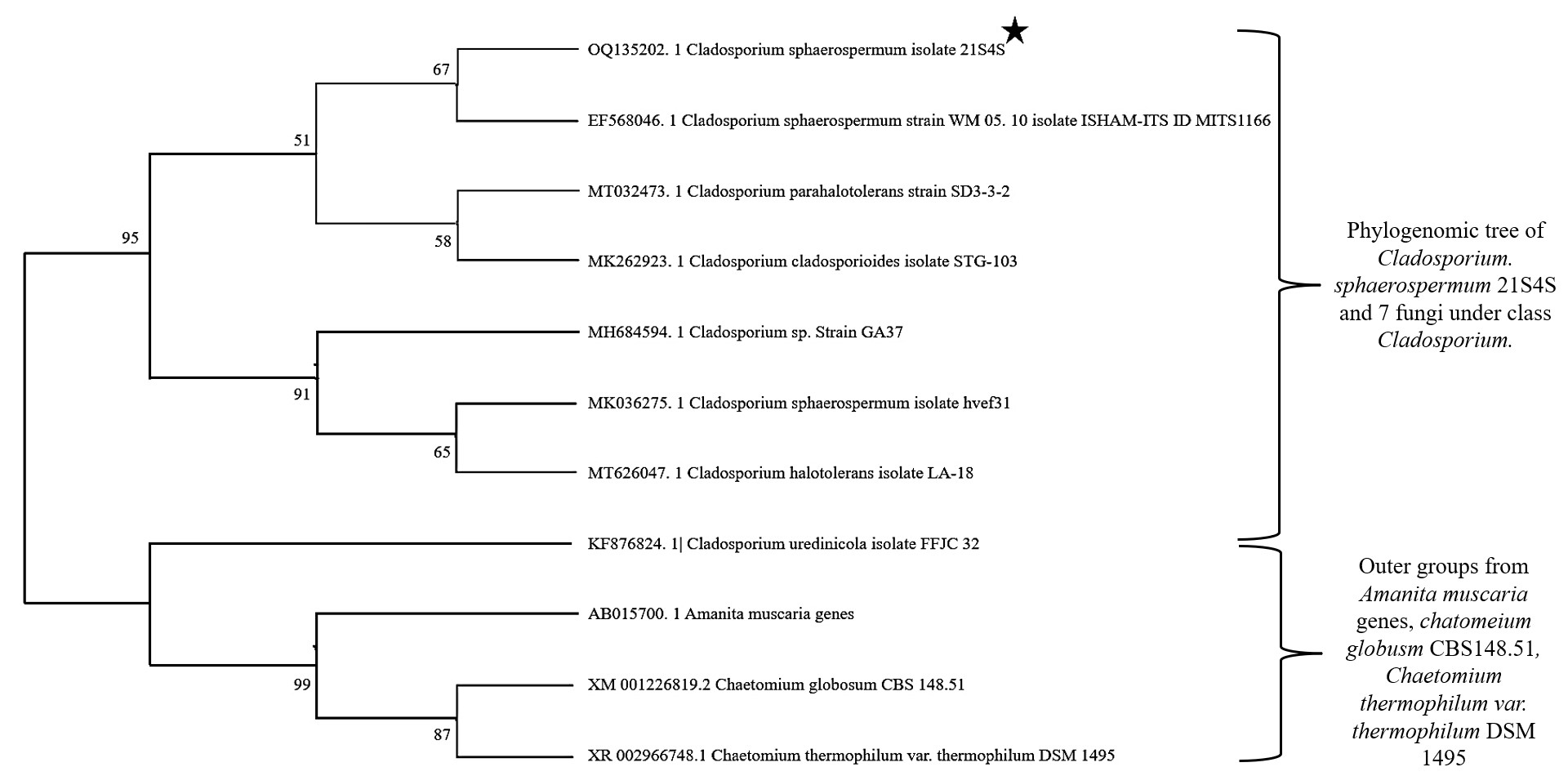


**Supplementary Figure S3.** Phylogeny tree of LDPE degrading C. sphaerospermum (indicated with asterik)

s


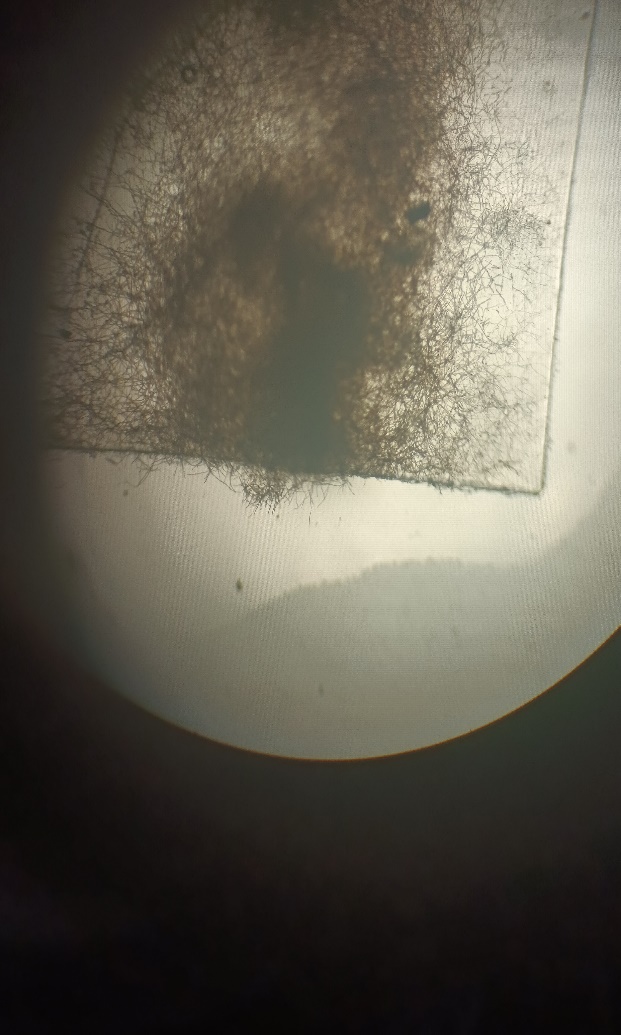


**Supplementary Figure S4:** Light Microscopic image of C. sphaerospermum on LDPE film
